# Correlations between structure and random walk dynamics in directed complex networks

**DOI:** 10.1063/1.2766683

**Published:** 2007-08-01

**Authors:** Luciano da Fontoura Costa, Olaf Sporns, Lucas Antiqueira, Maria das Graças Volpe Nunes, Osvaldo N. Oliveira

**Affiliations:** Instituto de Física de São Carlos, Universidade de São Paulo, P.O. Box 369, 13560-970, São Carlos, São Paulo, Brazil; Department of Psychological and Brain Sciences, Indiana University, Bloomington, Indiana 47405; Instituto de Física de São Carlos, Universidade de São Paulo, P.O. Box 369, 13560-970, São Carlos, São Paulo, Brazil; Instituto de Ciências Matemáticas e de Computação, Universidade de São Paulo, P.O. Box 668, 13560-970, São Carlos, São Paulo, Brazil; Instituto de Física de São Carlos, Universidade de São Paulo, P.O. Box 369, 13560-970, São Carlo, São Paulo, Brazil

## Abstract

In this letter the authors discuss the relationship between structure and random walk dynamics in directed complex networks, with an emphasis on identifying whether a topological hub is also a dynamical hub. They establish the necessary conditions for networks to be topologically and dynamically fully correlated (e.g., word adjacency and airport networks), and show that in this case Zipf’s law is a consequence of the match between structure and dynamics. They also show that real-world neuronal networks and the world wide web are not fully correlated, implying that their more intensely connected nodes are not necessarily highly active.

We address the relationship between structure and dynamics in complex networks by taking the steady-state distribution of the frequency of visits to nodes–a dynamical feature–obtained by performing random walks^1^ along the networks. A complex network^2–5^ is taken as a graph with directed edges and associated weights, which are represented in terms of the weight matrix W. The N nodes in the network are numbered as i=1,2,…,N, and a directed edge with weight M, extending from node j to node i, is represented as W(i,j)=M. No self-connections (loops) are considered. The *in* and *out strengths* of a node i, abbreviated as is(i) and os(i), correspond to the sum of the weights of its in- and outbound connections, respectively. The stochastic matrix S for such a network is

S(i,j)=W(i,j)∕os(j). (1)

The matrix S is assumed to be irreducible; i.e., any of its nodes can be accessible from any other node, which allows the definition of a unique and stable steady state. An agent, placed at any initial node j, chooses among the adjacent outbound edges of node j with probability equal to S(i,j). This step is repeated a large number of times T, and the frequency of visits to each node i is calculated as v(i)=(numberofvisitsduringthewalk)∕T. In the steady state (i.e., after a long time period T), v=Sv and the frequency of visits to each node along the random walk may be calculated in terms of the eigenvector associated with the unit eigenvalue (e.g., Ref. 6). For proper statistical normalization we set $\sum_{p}v\left(p\right)=1$. The dominant eigenvector of the stochastic matrix has theoretically and experimentally been verified to be remarkably similar to the corresponding eigenvector of the weight matrix, implying that the adopted random walk model shares several features with other types of dynamics, including linear and nonlinear summations of activations and flow in networks.

In addition to providing a modeling approach intrinsically compatible with dynamics involving successive visits to nodes by a single or multiple agents, such as is the case with world wide web (WWW) navigation, text writing, and transportation systems, random walks are directly related to diffusion. More specifically, as time progresses, the frequency of visits to each network node approaches the activity values which would be obtained by the traditional diffusion equation. A full congruence between such frequencies and activity diffusion is obtained at the equilibrium state of the random walk process. Therefore, random walks are also directly related to the important phenomenon of diffusion, which plays an important role in a large number of linear and nonlinear dynamic systems including disease spreading and pattern formation. Random walks are also intrinsically connected to Markov chains, electrical circuits, and flows in networks, and even dynamical models such as Ising. For such reasons, random walks have become one of the most important and general models of dynamics in physics and other areas, constituting a primary choice for investigating dynamics in complex networks.

The correlations between activity (the frequency of visits to nodes v) and topology (out strength os or in strength is) can be quantified in terms of the Pearson correlation coefficient r. For full activity-topology correlation in directed networks, i.e., ∣r∣=1 between v and os or between v and is, it is enough that (i) the network must be strongly connected, i.e., S is irreducible, and (ii) for any node, the in strength must be equal to the out strength. The proof of the statement above is as follows. Because the network is strongly connected, its stochastic matrix S has a unit eigenvector in the steady state, i.e., v=Sv. Since S(i,j)=W(i,j)∕os(j), the ith element of the vector Sos is given as

$$S\left(i,1\right)\mathrm{os}\left(1\right)+S\left(i,2\right)\mathrm{os}\left(2\right)+\cdots +S\left(i,N\right)\mathrm{os}\left(N\right)=\frac{W\left(i,1\right)}{\mathrm{os}\left(1\right)}\mathrm{os}\left(1\right)+\frac{W\left(i,2\right)}{\mathrm{os}\left(2\right)}\mathrm{os}\left(2\right)+\cdots +\frac{W\left(i,N\right)}{\mathrm{os}\left(N\right)}\mathrm{os}\left(N\right)=W\left(i,1\right)+W\left(i,2\right)+\cdots +W\left(i,N\right)=\mathrm{is}\left(i\right).$$ (2)

By hypothesis, is(i)=os(i) for any i and, therefore, both os and is are eigenvectors of S associated with the unit eigenvalue. Then os=is=v, implying full correlation between frequency of visits and both in and out strengths.

An implication of this derivation is that for perfectly correlated networks, the frequency of symbols produced by random walks will be equal to the out strength or in strength distributions. Therefore, an out strength scale-free^3^ network must produce sequences obeying Zipf’s law^7^ and vice versa. If, on the other hand, the node distribution is Gaussian, the frequency of visits to nodes will also be a Gaussian function; that is to say, the distribution of nodes is replicated in the node activation. Although the correlation between node strength and random walk dynamics in undirected networks has been established before^8^ (including full correlation^9,10^), the findings reported here are more general since they are related to any directed weighted network, such as the WWW and the airport network. Indeed, the correlation conditions for undirected networks can be understood as a particular case of the conditions above.

A fully correlated network will have ∣r∣=1. We obtained r=1 for texts by Darwin^11^ and Wodehouse^12^ and for the network of airports in the USA.^13^ The word association network was obtained by representing each distinct word as a node, while the edges were established by the sequence of immediately adjacent words in the text after the removal of stopwords^14^ and lemmatization.^15^ More specifically, the fact that word U has been followed by word V, M times during the text, is represented as W(V,U)=M. Zipf’s law is known to apply to this type of network.^16^ The airport network presents a link between two airports if there exists at least one flight between them. The number of flights performed in one month was used as the strength of the edges.

We obtained r for various real networks (Table I), including the fully correlated networks mentioned above. To interpret these data, we recall that a small r means that a hub (large in or out strength) in topology is not necessarily a center of activity. Notably, in all cases considered r is greater for the in strength than for the out strength. This may be understood with a trivial example of a node from which a high number of links emerge (implying large out strength) but which has only very few inbound links. This node, in a random walk model, will be rarely occupied and thus cannot be a center of activity, though it will strongly affect the rest of the network by sending activation to many other targets. Understanding why a hub in terms of in strength may fail to be very active is more subtle. Consider a central node receiving links from many other nodes arranged in a circle, i.e., the central node has a large in strength but with the surrounding nodes possessing small in strength. In other words, if a node i receives several links from nodes with low activity, this node i will likewise be fairly inactive. In order to further analyze the latter case, we may examine the correlations between the frequency of visits to each node i and the *cumulative hierarchical in and out strengths* of that node. The hierarchical degree^17–19^ of a network node provides a natural extension of the traditional concept of node degree. The immediate neighbors of a node i are called the first hierarchical level of i. The subsequent hierarchical levels are obtained as follows. The level h+1 contains the neighbors of the nodes of level h. The cumulative hierarchical out strength of a node i at the hierarchical level h corresponds to the sum of the weights of the edges extending from the hierarchical level h−1 to the level h, plus the out strengths obtained from hierarchy 1 to h−1. Similarly, the cumulative in strength of a node i at hierarchical level h is the sum of the weights of the edges from hierarchical level h to the previous level h−1, plus the in strengths obtained from hierarchy 1 to h−1. The traditional in and out strengths are, respectively, the cumulative hierarchical in and out strengths at hierarchical level 1 (see Supplementary Methods in Refs. 20 for an illustration of hierarchical levels). Because complex networks are also small world structures, it suffices to consider hierarchies up to two or three levels.

For the least correlated network analyzed, viz., that of the largest strongly connected cluster in the network of WWW links in the domain of Ref. 21 (Massey University, New Zealand) (Refs. 22 and 23) activity could not be related to in strength at any hierarchical level. Because the Pearson coefficient corresponds to a single real value, it cannot adequately express the coexistence of the many relationships between activity and degrees present in this specific network as well as possibly heterogeneous topologies. Very similar results were obtained for other WWW networks, which indicate that the reasons why topological hubs have not been highly active cannot be identified at the present moment (see, however, discussion for higher correlated networks below).

However, for the two neuronal structures of Table I that are not fully correlated (network defined by the interconnectivity between cortical regions of the cat^24^ and network of synaptic connections in *C. elegans*^25^), activity was shown to increase with the cumulative first and second hierarchical in strengths. In the cat cortical network, each cortical region is represented as a node, and the interconnections are reflected by the network edges. Significantly, in a previous paper,^26^ it was shown that when connections between cortex and thalamus were included, the correlation between activity and outdegree increased significantly. This could be interpreted as a result of increased efficiency with the topological hubs becoming highly active. Furthermore, for the fully correlated networks, such as word associations obtained for texts by Darwin and Wodehouse, activity increased basically with the square of the cumulative second hierarchical in strength (see Supplementary Fig. 2. in Ref. 20). In addition, the correlations obtained for these two authors are markedly distinct, as the work of Wodehouse is characterized by substantially steeper increase of frequency of visits for large in strength values (see Supplementary Fig. 3 in Ref. 20). Therefore, the results considering higher cumulative hierarchical degrees may serve as a feature for authorship identification.

In conclusion, we have established (i) a set of conditions for full correlation between topological and dynamical features of directed complex networks and demonstrated that (ii) Zipf’s law can be naturally derived for fully correlated networks. Result (i) is of fundamental importance for studies relating the dynamics and connectivity in networks, with critical practical implications. For instance, it not only demonstrates that hubs of connectivity may not correspond to hubs of activity but also provides a sufficient condition for achieving full correlation. Result (ii) is also of fundamental importance as it relates two of the most important concepts in complex systems, namely, Zipf’s law and scale-free networks. Even though sharing the feature of power law, these two key concepts had been extensively studied on their own. The result reported in this work paves the way for important additional investigations, especially by showing that Zipf’s law may be a consequence of dynamics taking place in scale-free systems. In the cases where the network is not fully correlated, the Pearson coefficient may be used as a characterizing parameter. For a network with very small correlation, such as the WWW links between the pages in a New Zealand domain analyzed here, the reasons for hubs failing to be active could not be identified, probably because of the substantially higher complexity and heterogeneity of this network, including varying levels of clustering coefficients, as compared to the neuronal networks.

## Figures and Tables

**Table I.** Number of nodes (No. nodes), number of edges (No. Edges), means and standard deviations of the clustering coefficient (CC), cumulative hierarchical in strengths for levels 1–4 (IS1–IS4), cumulative hierarchical out strengths for levels 1–4 (OS1–OS4), and the Pearson correlation coefficients between the activation and all cumulative hierarchical in strengths and out strengths $\left(r_{\mathrm{IS}1}–r_{\mathrm{OS}4}\right)$ for the complex networks considered in the present work.

	Cortex	*C. elegans*	Airports	Darwin	Wodehouse	WWW
No. nodes	53	191	280	3678	3705	10 810
No. edges	826	2449	4160	22 095	16 939	158 102
CC	0.60±0.15	0.22±0.11	0.62±0.41	0.04±0.11	0.03±0.08	0.60±0.21
IS1	25.89±9.42	100.82±110.03	2041.07±4323.33	7.87±22.15	5.29±16.15	14.63±155.87
IS2	217.13±56.68	1183.32±960.60	76068.88±53936.38	329.61±648.33	188.45±385.21	176.00±917.67
IS3	285.02±27.13	3543.97±1118.85	110381.09±35614.97	3352.93±2716.07	1977.58±1758.30	879.71±2635.18
IS4	285.68±27.13	4164.04±535.73	113662.07±32404.79	6943.53±2470.62	4830.73±1876.14	2468.12±4528.49
OS1	25.89±11.87	100.82±73.69	2041.07±4329.44	7.87±22.15	5.29±16.15	14.63±10.58
OS2	217.96±89.94	1156.76±675.14	76049.93±54196.34	313.16±626.72	187.60±394.19	176.00±131.02
OS3	296.98±34.93	3071.82±806.15	110771.60±35721.52	3234.23±2705.50	1961.32±1778.45	913.55±495.34
OS4	298.94±32.19	3532.41±473.59	114054.35±32493.50	6753.76±2454.90	4823.73±1853.97	2356.92±1200.37
$r_{\mathrm{IS}1}$	0.83	0.78	1.00	1.00	1.00	0.15
$r_{\mathrm{IS}2}$	0.58	0.84	0.33	0.86	0.82	0.09
$r_{\mathrm{IS}3}$	0.24	0.43	0.11	0.42	0.43	0.13
$r_{\mathrm{IS}4}$	0.24	0.35	0.08	0.20	0.22	0.11
$r_{\mathrm{OS}1}$	0.39	0.20	1.00	1.00	1.00	0.00
$r_{\mathrm{OS}2}$	0.30	0.01	0.33	0.87	0.81	−0.03
$r_{\mathrm{OS}3}$	−0.03	−0.19	0.11	0.42	0.43	−0.05
$r_{\mathrm{OS}4}$	−0.07	−0.33	0.07	0.20	0.22	−0.07
